# Analyzing clinical laboratory data outcomes in retrospective cohort studies using TriNetX

**DOI:** 10.11613/BM.2025.030502

**Published:** 2025-08-15

**Authors:** Joshua Wang, Kuo-Wang Tsai, Chien-Lin Lu, Kuo-Cheng Lu

**Affiliations:** 1 Department of Research, Taipei Tzu Chi Hospital, Buddhist Tzu Chi Medical Foundation, New Taipei City, Taiwan; 2 School of Medicine, Fu Jen Catholic University, New Taipei City, Taiwan; 3 Department of Internal Medicine, Fu Jen Catholic University Hospital, Fu Jen Catholic University, New Taipei City, Taiwan; 4 Department of Medicine, Taipei Tzu Chi Hospital, Buddhist Tzu Chi Medical Foundation, New Taipei City, Taiwan

**Keywords:** retrospective studies, clinical laboratory information systems, electronic health records

## Abstract

TriNetX, a rapidly growing global network of anonymized patient data, enables clinical researchers to perform large-scale retrospective cohort studies. However, its functionality for querying laboratory data outcomes is significantly constrained, as it only provides the results of the most recent test within a specified observation period. Consequently, the platform is not optimized for analyzing laboratory data collected at multiple time points during an observation period. This paper introduces innovative, data-informed solutions to address these limitations, offering practical guidance for researchers aiming to leverage TriNetX for examining clinical laboratory data.

## Graphical abstract

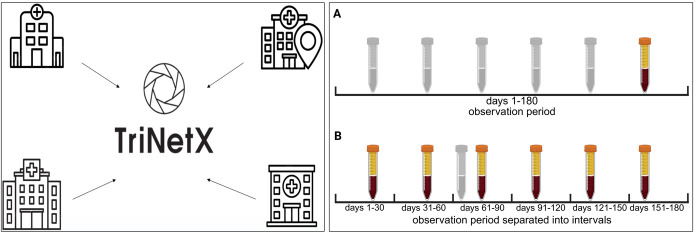

## Highlights

• TriNetX is a rapidly expanding data source for retrospective cohort studies

• The platform is currently not optimised for querying laboratory data

• We provide new solutions that may help researchers analyse laboratory data on TriNetX

## Introduction

Differences in laboratory test results between patient populations can provide important information on disease progress and the efficacy of therapies, ultimately helping to improve clinical decision making and patient outcomes (*1*-*3*). The TriNetX database enables users to query and analyze de-identified patient data sourced from numerous healthcare organizations (*4*). The extensive scale of this aggregated data facilitates robust retrospective cohort analyses, with hundreds of peer-reviewed studies already published utilizing the network (*5*). As of April 2025, the TriNetX Global Collaborative Network had electronic health record data for an estimated 179 million patients.

While TriNetX provides unprecedented statistical power to conduct retrospective cohort studies, analyses of the platform’s limitations are beginning to emerge (*6*). Methodological concerns have also been raised in letters to the editor submitted in response to TriNetX-based studies, and in the retraction statements of TriNetX-based studies (*7*-*9*). However, to the best of the authors knowledge no analysis of laboratory-based methods using the TriNetX platform by researchers external to the organisation has been performed. This paper therefore provides an independent overview of the laboratory test data analytical capabilities of the TriNetX platform. Key limitations with the platform will be highlighted, and novel workarounds will be proposed to enable medical laboratory scientists to better utilise the platform.

## An overview of TriNetX research design

Patient cohorts can be designed by defining sets of inclusion and exclusion criteria. In real-time, TriNetX queries all member healthcare organisations' anonymised electronic health records, and returns summary data to the users specifying the number of patients that meet the defined criteria (*4*). To analyse patient outcomes, a time window following the index event can be selected and the incidence of these outcomes for the patient cohort can be calculated. For comparative analyses of two patient cohorts, the platform enables 1:1 propensity score matching, leveraging a set of variables selected by the user and a specified time window prior to the defined index event. The risk ratios calculated from the outcome analysis can be used to identify particular variables associated with a clinical outcome.

TriNetX supports the querying of laboratory test results, which are structured and annotated according to Logical Observation Identifiers Names and Codes (LOINC) (*10*). Users can search for a laboratory test using either the test’s long common name, or its associated LOINC code (*11*). Laboratory tests can be used to define patient cohorts, acting as inclusion and exclusion criteria. They can also be queried as outcomes over a particular observation period. Searches can be performed for the presence or absence of a particular laboratory test (*e.g.* this cohort can only include patients that have had a serum selenium test). Laboratory test terms can also be filtered to only return test results within a particular range (*e.g.* this cohort can only include patients that have had a serum selenium test outcome above 150 ng/mL).

## General limitations of laboratory data on TriNetX

While TriNetX provides a streamlined approach to analysing large amounts of patient data, there are a number of limitations inherent to federated clinical data networks. Firstly, in order to remain compliant with the Health Insurance Portability and Accountability Act of 1996, access to raw patient data/laboratory values are not available to users. The platform instead only provides summary statistics in response to the user’s query parameters. Additionally, propensity score matching can only be performed between two patient cohorts, meaning that propensity-matched comparisons between three or more groups with different laboratory test results are not possible.

Laboratory tests belonging to the same LOINC code can still differ significantly in the laboratory method used. For method variance that results in laboratory values measured in non-standard units, TriNetX developed a successful unit harmonization procedure (*12*). However, this unit harmonization does not necessarily resolve underlying variance in laboratory methods within a LOINC code. Additionally, while laboratory data is sanitized to remove illogical values, the exact methods used to sanitize data for different laboratory tests are not disclosed. Therefore, test results with an identical LOINC code are not necessarily interchangeable when they are obtained from different laboratories due to differences in methods and protocols. Users should therefore consider the extent of methodological variance possible for laboratory tests that fall under a single LOINC code.

## Limitations with using laboratory data to define patient cohorts in TriNetX

**Figure 1:**
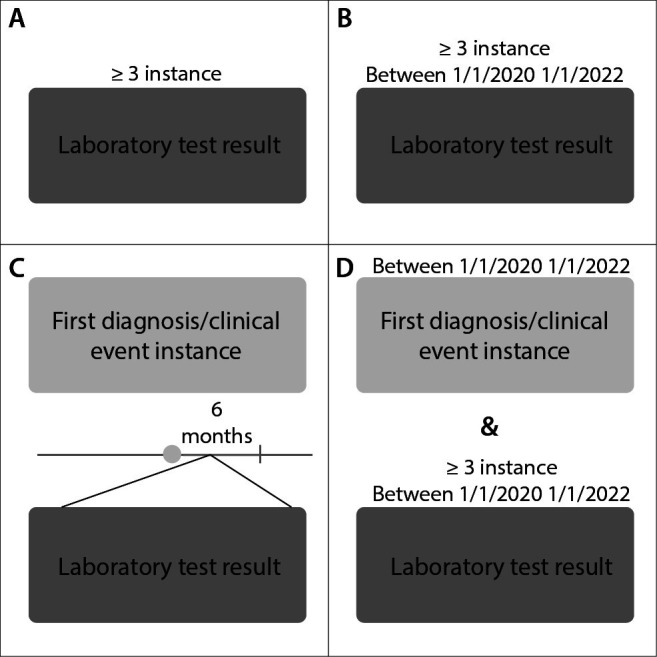
Cohort design in TriNetX using laboratory test results. A) patients with multiple instances of the same laboratory test result can be queried. B) patients with multiple instances of the same laboratory test result within a specified date range can be queried. C) patients with a single instance (not multiple instances) of a laboratory test result within a time period relative to a first instance of diagnosis can be queried. D) patients with multiple instances of the same laboratory test result within a specified date range, and a first instance of diagnosis within the same date range can be queried.

An important limitation at the cohort design stage is that it can be difficult to search for repeated laboratory test results within a specific timeframe (Figure 1). Repeated laboratory test results are important to differentiate between sustained abnormal laboratory values and natural fluctuations in solute concentrations. For example, if a researcher wishes to examine if vitamin D deficiency within the first 6 months of a patient’s first schizophrenia diagnosis influences mental health outcomes, then vitamin D deficiency may be defined as having three or more serum 25-hydroxyvitamin D (25(OH)D) test results below 20 nmol/L (Figure 1A). A timeframe can be added to this requirement (Figure 1B). However, if the researcher wishes to only include test results within a timeframe relative to the patient’s first schizophrenia diagnosis, a filter for multiple tests cannot be added (Figure 1C). Therefore, at best an identical timeframe could be placed on the schizophrenia diagnosis and three vitamin D deficient laboratory test results, which could include patients with vitamin D deficiency before a schizophrenia diagnosis as well as after (Figure 1D). Therefore, it is not always possible to directly query for repeated test results within a timeframe relative to another incident.

## Analysing laboratory test results as outcomes in TriNetX

**Figure 2:**
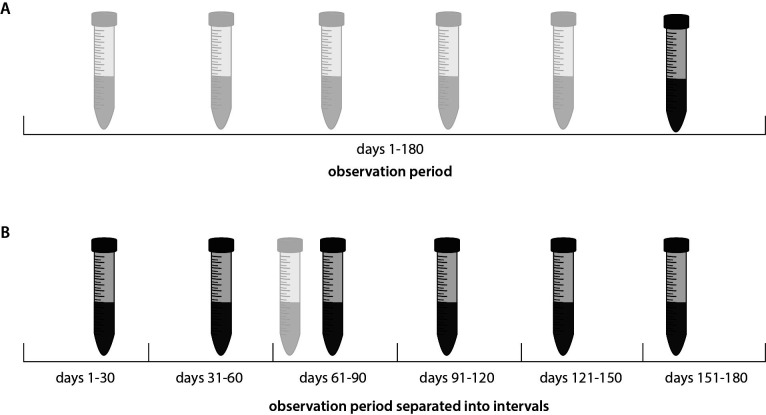
Analysing laboratory test results as a patient outcome in TriNetX. A) TriNetX only returns the most recent laboratory test result in a given outcome observation period, meaning that the results of previous test results in the observation period will not be considered. B) by splitting the outcome observation period into smaller intervals, more laboratory test results can be captured. However, some test results may still be missed due to variance in laboratory test frequency. Even if one-day intervals are used, TriNetX will still only return the most recent test result in a given day, excluding test result data from laboratory tests that are performed multiple times in one day.

Laboratory test results can also be queried as an outcome of interest in patient cohorts for a specified time-range following a pre-defined index event. However, the platform only provides the result of the most recent test instance within the specified outcome window. For instance, if hypoglycemia is queried as an outcome within a period of 1-180 days following an index event, and a patient records a positive test on day 97 but a negative test on day 135, the patient would be classified as not hypoglycemic (Figure 2A). This limitation renders the approach unsuitable for laboratory parameters that are frequently measured within the examined patient cohort (some examples are provided in Table 1). In this section we describe potential solutions to this limitation. Laboratory test results can be analyzed by dividing the observation period into distinct intervals (Figure 2B). Researchers can then manually combine the interval results to generate a dataset containing multiple laboratory test results *per* patient within the desired outcome period (*13*). The length of these intervals is determined by balancing two key factors. Firstly, the researcher must ensure that the observation period does not have more than one instance of testing for the laboratory outcome, thereby ensuring that all of the returned laboratory data for that interval is valid. For a given laboratory test result of interest, users can simultaneously query the number of instances of the laboratory test itself (*i.e.*, without a specified test result). This will provide an analysis of how many patients were tested more than once during this period. By extension, this data informs the researcher of the proportion of the data that is invalid.

**Table 1:** Example TriNetX analyses illustrating the limitations of analysing laboratory data outcomes

**Research question**	**Index event**	**Chosen outcomes**	**Outcome observation period**	**Is the analysis suitable to answer the research question?**
Are obese patients more or less likely to survive following tamoxifen chemotherapy?	The first instance of tamoxifen use	Death	1-180 days following the index event	Yes
Are obese patients more at risk of hypocalcemia following tamoxifen chemotherapy?	The first instance of tamoxifen use	Serum calcium < 2.1 mmol/L (LOINC 13959-2)	1-180 days following the index event	No - only the last serum calcium test in the observation period will be considered
What are odds of chronic kidney disease patients suffering a myocardial infarction following acute kidney injury?	Any instance of acute kidney injury occurring after diagnosis of chronic kidney disease	Acute myocardial infarction (ICD-10-CM I21)	1-30 days following the index event	Yes
How does proteinuria progress in chronic kidney disease patients following suffering acute kidney injury?	Any instance of acute kidney injury occurring after diagnosis of chronic kidney disease	Urine albumin/creatinine mass ratio in urine (LOINC 9318-7)	1-30 days following the index event	No - only the last urine albumin/creatinine ratio test in the observation period will be considered

Secondly, the researcher must ensure that there are enough patients with the specified test result in the time interval for TriNetX to return results. In order to maintain patient privacy, TriNetX does not report any outcomes with less than 10 patients, instead simply reporting the number of patients as “≤ 10”. Therefore, outcome periods that are split too finely risk returning unusable data. For researchers interested in laboratory outcomes that are either binary, or above/below a certain threshold, a potential workaround is to run the TriNetX analysis on the opposite test result. For example, if less than 10 patients in an outcome period had hypocalcemia (serum Ca < 2.1 mmol/L), then the outcome can instead be defined as all patients *without* hypocalcemia (serum Ca ≥ 2.1 mmol/L). Concurrently, the overall number of serum Ca tests during the outcome period can be queried, allowing the researcher to manually calculate the number of hypocalcemia patients in the outcome period. If this method is used, then short outcome intervals can be used as long as the number of tests performed during this period exceeds 20.

## Example laboratory test result analysis

To demonstrate this strategy, we have provided results from an example TriNetX-based analysis (Table 2). This analysis is based on a study the authors recently performed examining the effects of combination therapy on hyponatremia in respiratory and thoracic cancer patients (*13*). The analysis was performed on the Taiwan Global Collaborative Network. All data were collected on January 13, 2025 following Institutional Review Board approval (Approval Number: 13-IRB141).

**Table 2:** Most recent serum sodium (Na) test result of severe hyponatremia (serum Na < 125 mmol/L) 16,445 respiratory/thoracic cancer adult patients receiving immune checkpoint inhibitor and cisplatin combination therapy in the first 90 days following treatment

	**Analysis 1: 90-day interval**																
Outcome period	1-90																
Patients with outcome	426 (2.59%)																
Proportion of patients with invalid data (≥ 2 tests)	96%																
	**Analysis 2: 45-day intervals**																
Outcome period (days)	1-45									46-90							
Patients with outcome	309 (1.88%)									170 (1.03%)							
Proportion of patients with invalid data (≥ 2 tests)	89%									88%							
	**Analysis 3: 30-day intervals**																
Outcome period (days)	1-30				31-60							61-90					
Patients with outcome	258 (1.57%)				142 (0.86%)							110 (0.67%)					
Proportion of patients with invalid data (≥ 2 tests)	66%				60%							74%					
	**Analysis 4: 15-day intervals**																
Outcome period (days)	1-15		16-30				31-45		46-60				61-75			76-90	
Patients with outcome	156 (0.95%)		139 (0.85%)				82 (0.50%)		84 (0.51%)				67 (0.41%)			56 (0.34%)	
Proportion of patients with invalid data (≥ 2 tests)	62%		31%				28%		32%				27%			26%	
	**Analysis 5: 10-day intervals**																
Outcome period (days)	1-10	11-20		21-30		31-40		41-50			51-60			61-70	71-80		81-90
Patients with outcome	105 (0.64%)	102 (0.62%)		95 (0.58%)		44 (0.27%)		72 (0.44%)			53 (0.32%)			49 (0.30%)	41 (0.25%)		38 (0.23%)
Proportion of patients with invalid data (≥ 2 tests)	36%	27%		24%		30%		23%			21%			21%	26%		17%
Each analysis splits the observation period into smaller intervals, which reduces the number of patients with more than one test (invalid data) in that interval. Exact criteria used to define the patient cohorts in this analysis are available in the Supplementary Table 1.																	

The exact patient cohort design can be seen in Supplementary Table 1. In brief, only patients aged over 18 years of age were included. Patients must have had a diagnosis of a respiratory or intrathoracic cancer between January 1, 2011 and January 1, 2021. Patients with a diagnosis of primary adrenocortical insufficiency were excluded from the analysis as this disease could influence serum sodium (Na) concentrations. Additionally, within 6 months of cancer diagnosis, patients must have received both cisplatin/carboplatin and an immune checkpoint inhibitor. These criteria resulted in a patient cohort of 16,445 individuals.

To examine the prevalence of severe hyponatremia in this patient cohort in the 90 days following chemotherapy, the following outcome was examined: serum Na (LOINC 9029) result of < 125 mmol/L. The index event was defined as the first day where all inclusion criteria were met (*i.e.* the first day of combination therapy following the diagnosis of a respiratory/thoracic cancer). Additionally, the number of instances of serum Na tests was also queried.

As TriNetX only returns the most recent lab test results in a given observation period, any patients with more than one serum Na test in a given time period would have invalid test result data. Therefore, the same outcomes were ran against a titration of outcome period intervals (for example, days 1-45 and 46-90 for 45-day intervals; days 1-30, 31-60, 61-90 for 30-day intervals). For each interval, the number of patients with severe hyponatremia were recorded, alongside the percentage of patients with more than one serum Na test (*i.e*. invalid laboratory test results) in that interval.

## Limitations of TriNetX laboratory test result analysis

The results from our example analysis demonstrate that users attempting to analyse laboratory test results as a clinical outcome must split the outcome observation period into intervals small enough to avoid multiple tests, but large enough to ensure that data is not lost due to the censoring of identifiable patient outcome data. An important limitation even for daily outcome period intervals is that TriNetX will only provide the most recent test result of that day. It is therefore an unavoidable limitation that TriNetX cannot provide data on laboratory tests performed multiple times on the same day. Laboratory tests might be requested at a variable frequency both between and within patients. Therefore, splitting the observation period into equal intervals may not effectively reduce the proportion of invalid data during peak periods of testing. To account for this, users should also report the “number of instances” data alongside any laboratory test data, transparently demonstrating to readers the proportion of invalid data within time interval.

An important limitation to the above strategy is that it will likely include repeated tests results from the same patients. TriNetX does provide the option to “exclude patients with the outcome *prior* to the time window” when running outcomes, which would exclude repeated results. However, this strategy would decrease the number of patients ultimately returned with a laboratory test result of interest, increasing the likelihood of patient numbers dipping below 10 in a given outcome interval. In addition, this option also excludes all patients who have previously had the test at any point in their life prior to the outcome period. For common laboratory tests like a serum Na test, this would likely leave close to 0 patients available for analysis. Therefore, is likely that analysing laboratory test results as an outcome using the proposed strategy would include repeated patients. However, comparisons between patient cohorts with repeated patient data can still be made by performing Poisson regression modelling on the resulting concatenated data, allowing the researcher to detect if any statistically significant differences in laboratory outcomes between the populations exist (*14*).

## Conclusion

TriNetX provides a robust infrastructure for conducting large-scale retrospective cohort studies by querying electronic health records from participating healthcare organizations. However, the platform is not specifically optimized for analyzing laboratory test outcomes. To address this limitation, users can divide the outcome observation period into intervals. The length of these intervals should be carefully optimized to minimize the frequency of repeated tests within each interval while ensuring that the number of patients with the desired outcome exceeds 10. Patient outcome numbers can be further increased by including repeated measures in the concatenated data and by querying the more commonly observed outcome of a laboratory test. Utilising these strategies may better enable researchers to conduct retrospective cohort studies on laboratory outcomes of interest.

## Data Availability

All data generated and analyzed in the presented study are included in this published article.
